# Redox-Responsive Drug Delivery Systems: A Chemical Perspective

**DOI:** 10.3390/nano12183183

**Published:** 2022-09-14

**Authors:** Heba F. Abed, Waad H. Abuwatfa, Ghaleb A. Husseini

**Affiliations:** 1 Department of Biology, Chemistry and Environmental Sciences, American University of Sharjah, Sharjah P.O. Box 26666, United Arab Emirates; 2 Department of Chemical Engineering, College of Engineering, American University of Sharjah, Sharjah P.O. Box 26666, United Arab Emirates; 3 Materials Science and Engineering Program, College of Arts and Sciences, American University of Sharjah, Sharjah P.O. Box 26666, United Arab Emirates

**Keywords:** drug delivery systems, redox-responsive, reducing, linkers, bonds

## Abstract

With the widespread global impact of cancer on humans and the extensive side effects associated with current cancer treatments, a novel, effective, and safe treatment is needed. Redox-responsive drug delivery systems (DDSs) have emerged as a potential cancer treatment with minimal side effects and enhanced site-specific targeted delivery. This paper explores the physiological and biochemical nature of tumors that allow for redox-responsive drug delivery systems and reviews recent advances in the chemical composition and design of such systems. The five main redox-responsive chemical entities that are the focus of this paper are disulfide bonds, diselenide bonds, succinimide–thioether linkages, tetrasulfide bonds, and platin conjugates. Moreover, as disulfide bonds are the most commonly used entities, the review explored disulfide-containing liposomes, polymeric micelles, and nanogels. While various systems have been devised, further research is needed to advance redox-responsive drug delivery systems for cancer treatment clinical applications.

## 1. Introduction

As cancer continues to affect over 18 million lives yearly, causing over 9.8 million mortalities worldwide as of 2020, there is an urgent need to find a cure [1]. Numerous cancer therapies have been and continue to be researched and developed. These include classical therapies, such as chemotherapy; radiotherapy; and surgery, and more modern, local therapies, such as focused ultrasound and specific drugs targeting tumor cells [2]. Although widely used, conventional methods like chemotherapy are limited due to their toxic side effects, low specificity, and resistance to the treatment, whether inherently present or developed later after successful initial treatments [3]. Hence, alternative treatments with reduced side effects have been explored, such as smart drug delivery systems (SDDSs).

SDDSs are generally defined as particles with a typical size between 1–500 nm that can carry one or multiple therapeutics either by encapsulating them in a matrix via covalent linkages, adsorption, or simple dispersion; these agents are later released at the target site [4,5]. These SDDSs have emerged as promising anti-tumor agents due to their enhanced solubility, bioavailability, and targeting; their ability for controlled delivery; and their potential to reduce drug resistance [3,6]. Furthermore, these drug delivery systems have the added advantage of allowing for the simultaneous delivery of drugs and genes (co-delivery), allowing for synergistic effects and enhanced anti-tumor activity [7,8]. Therefore, these delivery systems have a greater preference over conventional anticancer therapies due to their more specific and less toxic characteristics.

**Figure 1:**
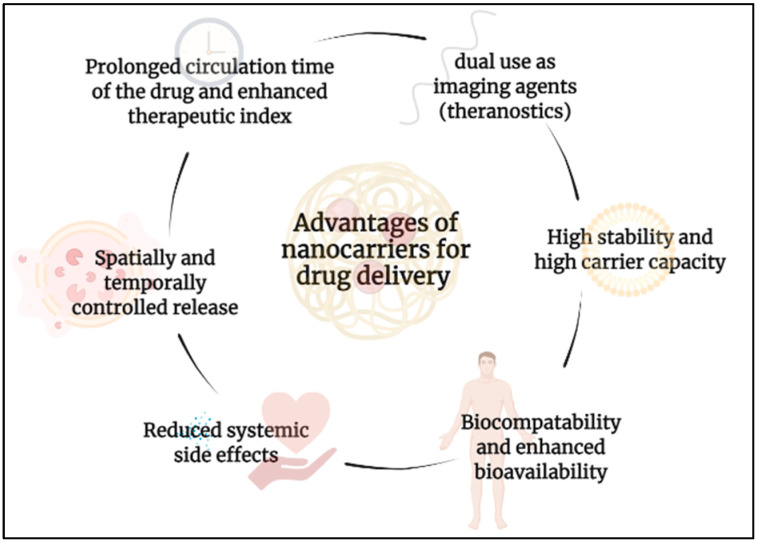
Advantages of using nanoparticles as nanocarriers in SDDSs.

Different types of smart drug delivery vehicles, generally referred to as nanocarriers, have been developed. Such nanoparticles include liposomes [4,9,10,11], micelles [12], dendrimers [13], quantum dots [14], metal organic frameworks (MOFs) [15], polymeric systems [16], and many other nanoparticles. Each class is distinct in its properties, with different advantages and limitations. Figure 1 summarizes the most important advantages of using nanoparticles as nanocarriers for controllable drug delivery. Furthermore, these nanoparticles have been manipulated and designed to be sensitive to external stimuli, allowing for targeted drug delivery at the tumor site [17]. Such external stimuli include heat [18], light [17,19], ultrasound waves [20], and magnetic fields [21,22]. Because the tumor microenvironment differs greatly from that of the surrounding healthy tissue, drug delivery nanocarriers have also been tailored to respond to internal stimuli specific to tumor cells. Some internal stimuli utilized include pH and enzymes [23], reactive oxygen species (ROS) [24,25,26], hypoxia (oxygen deficiency) [27], and the reductive environment of tumors [28,29]. These stimuli can be used alone or in various hybrid combinations for more effective drug delivery [30,31,32]. All these stimuli allow for enhanced targeting and drug release, improving the anti-tumor activity of drug delivery nanocarriers.

In recent years, redox-responsive DDSs, nanocarriers sensitive to the reducing environment of tumors, have progressively advanced, becoming increasingly promising as a potential cancer treatment platform. This is because of their distinct advantages, such as their enhanced biological stability, lower cytotoxicity, rapid cargo release, and greater overall therapeutic effects due to increased intracellular drug release [33]. Hence, this paper will herein review the fundamental principles, mechanisms, and chemical design of redox-responsive drug delivery systems used for cancer treatment.

## 2. Tumor Microenvironment and Reduction Mechanism

The microenvironment of tumor tissues is a highly reducing environment (also referred to as a reductive environment) as compared to surrounding healthy cells. The main biochemical that causes this distinctive reducing nature is glutathione (GSH), a tripeptide containing a thiol (-SH) group from the cysteine residue [17,34]. Intracellular GSH levels range from 1–10 mM in normal cells, while extracellular GSH levels are much lower (2–20 µM); however, GSH concentrations in tumor cells are over four times greater than in healthy cells [17,34,35]. This large discrepancy in GSH levels between tumor and healthy cells allows for the utilization of the reducing environment as an internal stimulus for drug delivery systems [36]. Additionally, over 80% of GSH molecules are concentrated in the cytosol, while other organelles contain lower amounts (~10%); this is beneficial as it facilitates the release of anti-tumor agents directly into the cytosol, where these agents act more effectively at their targets [36]. Therefore, intracellular GSH levels can be used as an internal stimulus for targeted drug delivery.

**Figure 2:**
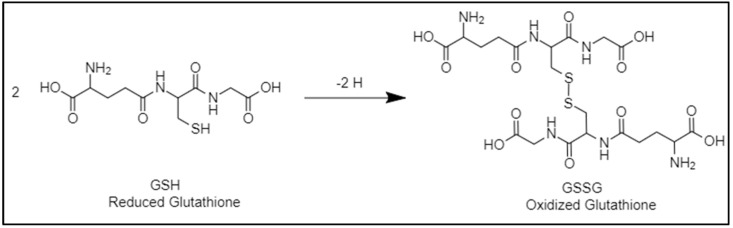
GSH/GSSG redox couple chemical structure.

The mechanism of action of GSH as a reducing agent involves the donation of a hydrogen atom from the thiol group to specific chemical entities in the drug delivery nanocarrier [37]. Redox-responsive chemical entities incorporated in nanocarriers are often bonds that, upon receiving a hydrogen atom (reduction), will undergo a conformational change, leading to breakage and gap in the nanocarrier’s structure [38,39]. Hence, when redox-responsive drug nanocarriers are exposed to the high GSH concentration at tumor sites, they will receive hydrogen atoms from GSH that will lead to substantial breakages in the nanocarrier, causing it to disintegrate and release the loaded anti-tumor agents into the cell. Simultaneously, GSH is oxidized to glutathione disulfide (GSSG) by forming a disulfide bond, as a result of the donation of hydrogen [37]. The nature of the disintegration of the nanocarrier is dependent on the type of the nanocarrier, the location of the redox-sensitive chemical entities, and the type of redox-sensitive chemical entities present. Through this process, the reducing environment of tumors can easily and rapidly facilitate nanocarrier disintegration and targeted cargo release. The GSH/GSSG redox couple chemical reaction is depicted in Figure 2.

Another biomolecule that contributes to the reducing potential of tumor cells is NADPH and its oxidized state NADP^+^. However, NADPH levels in cells are much lower than GSH levels, and thus NADPH contributes less than GSH in stimulating redox-responsive nanocarriers [40]. Similarly, while contributing to the overall reductive environment of tumors, the redox couple thioredoxin and thioredoxin reductase contribute less to triggering redox-responsive nanocarriers [40,41]. The gamma-interferon-inducible lysosomal thiol (GILT) reductase enzyme has also been found to contribute to the reducing potential of tumor cells through lysosomal actions; however, this is minimal compared to the GSH contribution [42,43]. Hence, the high reducing potential of tumor microenvironments is mainly due to elevated GSH levels, which allows for targeted drug delivery.

## 3. Chemical Design of Redox-Responsive Drug Delivery Systems

To utilize the reducing environment of tumor cells for targeted drug delivery, redox-sensitive chemical entities must be incorporated into the design of the drug delivery systems. Ideally, these chemical linkers would be reduced by GSH, leading to the disintegration of the nanocarrier or a morphological change in the nanocarrier’s structure that allows for efficient release of the loaded anti-tumor therapeutic agents [38,39]. Different functional groups utilized in nanocarriers include disulfide bonds (–S–S–) [38,39,44], diselenide bonds (–Se–Se–) [45,46], succinimide-thioether linkages [8,28,47,48,49,50,51,52], tetrasulfide bonds (–S–S–S–S–) [53,54,55,56], and platin conjugation (–Pt–) [30]. These chemical groups have been incorporated into different types of nanocarriers to create effective drug delivery systems.

### 3.1. Disulfide Bonds

Disulfide bonds (S–S), covalent bonds between two sulfur atoms, are the most widely researched linkers used in the design and synthesis of SDDSs for cancer treatment. This is because they are susceptible to GSH and are easily and rapidly reduced to thiol groups in their presence, via a “thiol-disulfide exchange” reaction. In detail, the thiol-disulfide exchange redox reaction involves the donation of two hydrogen atoms by surrounding GSH molecules to the sulfur atoms of the disulfide bond, leading to the dissociation of the disulfide bond to form two thiol groups (–SH) [reduction] and the simultaneous oxidation of GSH to GSSG [oxidation] [57]. Hence, disulfide bonds are frequently used as linkers or crosslinkers in nanocarriers because, upon exposure to GSH at the tumor site, the disulfide bond will break, leading to complete disintegration of nanocarriers and effective cargo release [33,58]. Additionally, the use of disulfide bonds as linkers is favored because they are highly stable during blood circulation and will only break down when entering the tumor environment, thereby facilitating targeted delivery of chemotherapeutics to the tumor site alone and minimizing unwanted side effects [57]. Disulfide groups have been incorporated into different types of nanocarriers, and these will be explored in further detail in a later section.

**Figure 3:**
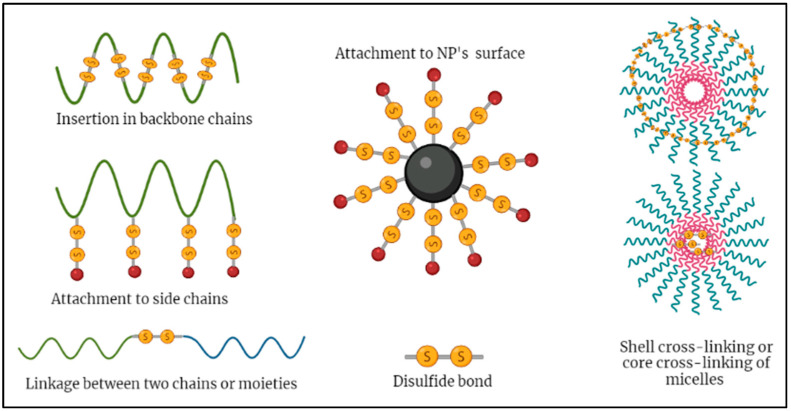
Disulfide bond locations in SDDSs.

Moreover, the position of disulfide linkers in the drug delivery nanocarriers greatly influences the stability and effectiveness of the drug delivery system, allowing for the flexible design of various redox-responsive nanocarriers. Disulfide linkers have been utilized in the backbone of polymers used in the synthesis of nanocarriers [39,59]. Furthermore, they have also been used as side chain linkers [60] and as linkers on the surface of nanoparticles [61]. Disulfide linkers have also been used widely to link two chemical moieties, i.e., in making copolymers that can later aggregate and fold into micelles [32,44,62,63]. Additionally, disulfide bonds have been extensively used as crosslinkers in nanogels [64] and micelles, where, in micelles, the disulfide bonds were used to crosslink the inner core [38,65,66,67] or outer shell [68,69] of polymeric micelles. Figure 3 summarizes the different positions of disulfide linkers in SDDSs.

### 3.2. Diselenide Bonds

Diselenide bonds are covalent bonds between two selenium atoms (Se–Se); when reduced, two selenol (–SeH) groups form. This reduction occurs via the donation of a hydrogen atom from GSH to each selenium atom in the bond, leading to bond cleave and –SeH formation. Selenium and sulfur atoms are very similar in chemistry, as both belong to the same chemical group in the periodic table [29]. However, because selenium atoms are bigger than sulfur atoms, the diselenide bonds and carbon-selenium bonds (C–Se) are of lower dissociation energy and less stable than disulfide bonds, due to the longer bond length. While this characteristic of diselenide bonds is excellent when exposed to the reducing environment of tumors, as it enhances drug release, it also means that diselenide bonds have lower stability and can lead to the leakage of drugs when incorporated into a drug delivery system due to disintegration during circulation [29,33]. Diselenide-containing materials have also been known to have poor solubility, which hinders the effectiveness of diselenide-containing DDSs [70,71]. Nonetheless, diselenide bonds have excellent sensitivity to the reducing environments of tumor tissues (GSH) and have been utilized to develop redox-responsive drug delivery systems.

Diselenide bonds have been incorporated into different nanocarriers for efficient and effective drug delivery. Generally, diselenide bonds are used as linkers in the backbone of polymers that can later self-assemble into micelles [45,46,72,73,74,75], aggregate into stable nanoparticles [29,70], or form hydrogels [76,77] that can all deliver anti-tumor agents. To elaborate further, the diselenide bonds in the backbone could be within or between the polymer chains, as done in [46,77]. In [46], a polyurethane segment containing diselenide was polymerized with PEG [PEG–PESeSe–PEG] to form stable micelles. However, diselenide bonds can also be used as linkers between different polymer segments to form a block copolymer, as was demonstrated in [75,76]. In [75], 2 methoxypoly(ethylene glycol) (PEG) segments were linked to both ends of a polycaprolactone (PCL) segment via diselenide bonds to form a redox-sensitive triblock copolymer [CH_3_–PEG–SeSe–PCL–SeSe–PEG–CH_3_] that assembled into micelles which effectively encapsulated and delivered DOX.

In addition to their role as linkers, diselenide bonds have also been used as crosslinkers in the development of redox-sensitive micelles [78], hydrogels [79], and nanogels [80]. In [78], PEG-*b*-PBSe block copolymers were irradiated with visible light during self-assembly to induce the formation of Se–Se crosslinking bonds in the micelle’s core and simultaneously encapsulate DOX and camptothecin (CPT). The resulting crosslinked micelle was biocompatible and demonstrated no significant side effects, making them potential candidates for clinical applications [78]. Moreover, selenocystamine molecules (containing –SeH) bonds were used to crosslink *N*-hydroxysuccinimide modified PEG molecules via the formation of Se–Se bonds to form injectable, redox-sensitive hydrogels for effective drug delivery [79]. Meanwhile, poly(2-methacryloyloxyethyl phosphorylcholine) (PMPC) based nanogels were crosslinked with Se–Se containing *N,N′*-bis(acryloyl) selenocystamine (BMASC) via a reflux precipitation polymerization reaction to form a stable and biocompatible GSH-responsive DDS [80]. Additionally, carboxymethyl chitosan-based nanoparticles have been crosslinked with 3,3′-diselenodipropionic acid di(*N*-hydroxysuccinimide ester) (DSeDPA-NHS) to form redox-responsive DDSs that could effectively load DOX and deliver it in-vitro and in-vivo [81]. Thus, diselenide bonds are also used as crosslinkers in the design of redox-responsive DDSs.

An emerging type of redox-responsive nanocarrier that utilizes diselenide bonds are hybrid, mesoporous silica-based nanoparticles [82,83]. Mesoporous silica nanovehicles have been embedded with nanoparticles, coated with a protein gate of myoglobin or serum albumin via diselenide bonds, and loaded with DOX [82]. Upon exposure to the GSH, the diselenide bonds were cleaved, and DOX was released; additionally, the DDS was activated by pH and H_2_O_2_ in the tumor environment by changes in the protein conformation, releasing DOX [82]. Meanwhile, An et al. used Se–Se bonds in a silane coupling agent to clog the mesoporous silica pores and trap DOX in the developed hybrid drug carrier [83]. Both DDSs were biocompatible and demonstrated excellent cytotoxicity toward cancer cells [82,83]. Hence, diselenide bonds have been proven efficient in developing various redox-responsive DDSs.

Moreover, diselenide bonds are versatile in that they not only respond to the reducing environment of the tumor microenvironment (GSH) but can also be cleaved upon exposure to reactive oxygen species (ROS), which are generally present at higher concentrations (up to 1 mM) in tumor microenvironments as compared to normal cells [75,82]. In this case, the diselenide bonds would be oxidized into selenic acids (RSeOOH) [75]. Common ROSs include hydroxyl radicals (OH•), hydroperoxy radicals (•HO_2_), and hydrogen peroxide (H_2_O_2_), which is the reagent commonly used for in vitro experimentations [46,75,77,82]. Numerous studies have investigated the additional effect of the oxidative cleavage of redox-responsive DDSs containing diselenide bonds and whether it can be used as an additional internal stimulus to enhance tumor-targeted drug delivery [46,75,76,77,80,81,82,84]. In general, most of the designed, diselenide-containing DDSs disintegrated in the presence of varying concentrations of H_2_O_2_, with higher concentrations leading to quicker degradation and drug release at the tumor site [46,76,77]. When comparing the efficiency of oxidative cleavage via H_2_O_2_ against reductive cleavage via GSH, Yan et al. [82] found that the drug release from protein-gated nanoparticles in the presence of H_2_O_2_ and GSH was similar at equivalent concentrations of 1 mM. However, GSH concentrations in tumor environments are generally higher than H_2_O_2_ (up to 10 mM); thus, reductive cleavage of diselenide bonds is often the dominant cleavage taking place [34]. This is supported by various studies in which diselenide bond cleavage was significantly faster in the presence of GSH as compared to H_2_O_2_, although both were effective, with release percentages reaching up to 87% [75,80,81,84]. Therefore, diselenide bonds are useful chemical bonds that can be incorporated into various SDDSs for enhanced delivery via redox-responsiveness in the presence of GSH and ROS.

### 3.3. Succinimide-Thioether Linkages

**Figure 4:**
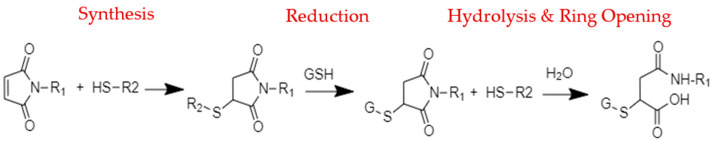
Succinimide-thioether linkage synthesis, reduction, and hydrolysis in tumor microenvironments.

Succinimide-thioether linkages, also known as maleimide thioether linkages, are a much more complex reduction-sensitive chemical entity when compared to disulfide and diselenide bonds. They are formed via the Michael addition reaction of maleimides with thiol groups (Figure 4). [28,47,48,49,85]. Upon exposure to tumor GSH concentrations, these succinimide-thioether groups undergo a retro Michael addition where the double bond is restored in the maleimide moiety and the thiol group is released (due to receiving a hydrogen atom from GSH). This is followed by a thiol exchange reaction where the thiol group in GSH attacks the double bond in the maleimide and forms a new succinimide-thioether [47,48]. The cleavage of the initial succinimide thioether linkage causes a gap in the nanocarrier structure, leading to subsequent disintegration and cargo release due to the reducing environment of tumors [48]. Hydrolysis may also occur, either initially or following the retro-Michael reaction and thiol exchange, leading to ring opening of the maleimide and more holes in the nanocarrier structure which can enhance cargo release [48]. It is important to note that only aryl thiol-based succinimide thioether linkages are GSH-sensitive, while alkyl thiols are not and will remain intact when exposed to GSH [8]. Furthermore, mechanistic studies investigating the GSH-susceptibility of succinimide thioether bonds found the type of thiol and *N*-substituent used to play key roles in the rate of cleavage of succinimide thioether linkages in reducing environments, where *N*-substituents capable of forming more hydrogen bonds and thiol groups of low pKas led to greater rates of bond cleavage in GSH [48]. Thus, the functional groups in the succinimide thioether linkages can be designed and modified to tune the reactivity and speed of release of cargo from DDSs containing these linkages. When compared to disulfide bonds, DDSs containing succinimide thioethers have been found to have release rates 10–100 times slower than those of disulfides but a longer circulation time, greater stability, and a more sustained drug release profile [47]. As such, succinimide thioether linkages are much more relevant in biomedical applications that require slower degradation profiles with prolonged drug delivery [47,48].

Research on succinimide thioether bonds in redox-responsive DDSs is much less common than disulfide and diselenide bonds, with fewer papers in the literature. Succinimide thioether bonds have most commonly been incorporated as crosslinkers in hydrogels [49,50,51,52] but have also been used in micelles [28] and hybrid hydrogel-liposome systems [8]. Hydrogels incorporating succinimide thioether linkages often utilized PEG as the building block polymer; however, different groups were used for PEG functionalization to form the succinimide thioether cross-linkages [49,50,51,52]. Baldwin et al. [49] formed hydrogels using thiolated-four-armed PEG polymers and crosslinked them with maleimide-functionalized heparin via the click Michael addition reaction. PEG was thiolated with either 3-mercaptopropanoice acid (MP), 4-mercaptophenylpropanoic acid (MPP), 3-mercaptoisobutyric acid (MIB), or 2,2-dimethyl-3-(4-mercaptophenyl)propionic acid (DMMPP), with MPP- and DMMPP-modified PEG-based hydrogels exhibiting high GSH-sensitivity while MP- and MIB-modified PEG-based hydrogels exhibiting significantly lower GSH sensitivity because of the absence of a retro-Michael cleavage as a result of the nonaromatic nature of the thiol groups. Subsequently, the MPP-PEG and DMMPP-PEG-based hydrogels were best at delivering low-molecular-weight heparin upon GSH exposure and hydrogel degradation, where MPP-PEG had the fastest heparin release profile [49]. These hydrogels are predicted to have anti-tumor properties; however, their capabilities have not been tested thoroughly. Similarly, a series of studies by Kharkar et al. developed several injectable hydrogels that consisted of multi-arm PEGs functionalized with aryl thiols and maleimide-functionalized PEGs or maleimide-functionalized heparin [50,51,52]. Aryl-thiol modifications have been done using moieties such as 4-mercaptophenylacetic acid (MPA) with additional ester modification [50,51] and MPA modified with photodegradable (PD) moieties [52] to allow for dual redox and light sensitivity. All hydrogels portrayed excellent GSH sensitivity at conditions mimicking in vivo GSH levels, with the hydrogels developed in [52], synthesized from a mix of PD-MPA-modified PEG, maleimide-modified PEG, and maleimide-modified heparin, exhibiting excellent delivery and release of bioactive proteins in vitro and in vivo due to the dual redox and light responsiveness of the hydrogels.

In addition to hydrogels, succinimide thioether bonds have also been utilized to develop a hybrid hydrogels-liposome DDS, where the liposomes were functionalized with maleimides and used as crosslinkers by reacting with thiolated, 4-armed PEG molecules [8]. The PEG polymers were modified with 4-mercaptohydrocinnamic acid as the source of aryl thiols, and the resulting hydrogel showed minimal degradation (~15%) at 10µM GSH and a significant, rapid degradation (~97%) at 10 mM concentrations of GSH within seven days. Meanwhile, hydrogels with no succinimide-thioether crosslinkers showed no significant degradation in both GSH conditions. Moreover, these hydrogels were effective for the delivery of DOX alone and the co-delivery of DOX and cytochrome C, allowing for synergism and greater anti-tumor efficacy through a two-stage release process [8]. Furthermore, succinimide thioether linkages were also incorporated in the preparation of micelles for the delivery of the fluorescent probe, Nile Red [28]. Succinimide thioether bonds were used as linkers in the copolymers by reacting arylthiol-modified xyloglucan oligosaccharides with maleimide-modified polycaprolactone polymers, where arylthiol modification was performed using an alkyl thiol. Since an alkyl thiol was used, the observed cleavage and drug release in the presence of GSH were relatively slower. As such, the authors proposed reacting bromomaleimide with thiols, instead of maleimides, which exhibited faster degradation when exposed to GSH due to the different mechanism [28]. From this, one may suggest using bromomaleimide-thiol linkages when interested in using alkyl thiols to overcome the previous restriction regarding the use of aryl thiols when forming succinimide thioether linkages for GSH-sensitivity. Nonetheless, succinimide thioether bonds are valuable, redox-sensitive moieties for the design of DDSs where slow and sustained drug release is preferred.

### 3.4. Tetrasulfide Bonds

Tetrasulfide bonds consist of four sulfur bonds linked to each other via covalent linkages (S–S–S–S). Although similar to disulfide bonds, tetrasulfide bonds are less commonly used in the synthesis of redox-responsive DDSs and have only been recently introduced [53,54,55,56]. When exposed to GSH, tetrasulfide bonds undergo multiple thiol-disulfide exchange reactions to eventually degrade into thiol groups (–SH) and produce hydrogen disulfide (H_2_S) [55,86]. The initial attachment of GSH can either be on the α or β sulfur atom, leading in either case to the cleavage of the tetrasulfide bond and degradation of the nanocarrier [86]. Furthermore, the generated H_2_S can play a role in killing cancer cells by damaging their mitochondria and reducing cellular respiration [56]. Due to these great properties, tetrasulfide bonds have been utilized in the development of redox-responsive DDSs.

The most common nanocarriers that tetrasulfide bonds have been incorporated in are mesoporous organosilica nanoparticles (oMSNs) [53,54]. Tetrasulfide bonds are generally introduced into oMSNs using bis-[gamma-(triethoxylsilicon)propyl]tetrasulfide as the S–S–S–S-containing reagent [53,54,55]. For example, Song et al. developed a surfactant-free Stöber method to synthesize oMSNs containing tetrasulfide bridges in the silesquioxane framework [54]. The oMSN had good GSH sensitivity, with significant degradation observed after incubation in 5 mM GSH for 60 h. However, the rate of degradation was highly variable across the oMSNs due to unequal interactions with GSH because of the relatively high hydrophobicity of the oMSNs. Furthermore, the synthesized oMSN was successful in encapsulating methylene blue and curcumin (separately) [54]. Hence, these oMSNs have the potential to be used for cancer applications. Wang et al. [53] also developed oMSNs containing different concentrations of tetrasulfide bonds to induce redox responsiveness in the nanocarriers and to study the effect of the tetrasulfide content on the nanocarrier’s properties. Volume ratios of tetraethylorthosilicate (TEOS, the silica source) to BTEPTS (the S–S–S–S source) studied were 4:1, 2:1, and 1:1, with total volumes being constant. While one would assume that the greater amount of tetrasulfide bonds would lead to faster degradation due to more reduction-sensitive bonds, the study found oMSNs formulated at a 2:1 TEOS:BTEPTS ratio had the fastest degradation rate (in 5 mM DTT) and oMSNs with 1:1 TEOS:BTEPTS ratios had the slowest degradation rate, which was even slower than oMSNs made with no S–S–S–S bonds. These findings are quite significant and indicate the importance of using an optimal content of tetrasulfide bonds, as excess tetrasulfide bonds can lead to lower surface area oMSNs and slower degradation rates [53]. Further studies are needed to investigate their efficiency in vitro and in vivo.

In addition to being incorporated in oMSNs alone, tetrasulfide bonds have been utilized in the development of hybrid oMSN nanocarriers, where oMSNs are mixed with other nanoparticles [55,56]. Song et al. [55] developed a core–shell hybrid nanocarrier, with gold nanorods (GNR) being the core structure and oMSNs containing tetrasulfide bonds being the shell. The oMSN coat was successful in encapsulating DOX in its mesopores, which was released rapidly upon GSH exposure due to the cleavage of tetrasulfide bonds. Meanwhile, the gold nanorod core was utilized for photothermal therapy and synergetic tumor eradication with DOX. The hybrid GNR-oMSN nanocarrier had excellent biocompatibility, rapid degradation in 10 mM GSH, and significant tumor inhibition in vivo, with no significant side effects—serving as a promising redox-responsive DDS for cancer treatment [55]. On the other hand, Liu et al. [56] developed a hybrid dendritic mesoporous organosilica (DMOS) DDS containing tetrasulfide bonds and doped with Mn^2+^ ions, Fe^3+^ ions, or Co^2+^ ions. The Mn^2+^-doped DMOS was effective in loading indocyanine green at high loading capacities while also being highly GSH-sensitive with rapid degradation rates and H_2_S generation. This led to significant apoptosis of cancer cells due to the redox-triggered release of indocyanine green and subsequent photothermal effect by irradiating indocyanine green with near-infrared radiation [56]. Hence, tetrasulfide bonds exhibit good sensitivity to GSH and can be used in the development of redox-responsive DDSs. Further studies are needed to explore the use of these tetrasulfide moieties in other types of nanocarriers, such as micelles, liposomes, nanogels, and others.

### 3.5. Platin Conjugation

Platin conjugation involves the integration of platinum atoms into the structure of the drug delivery system. In general, octahedral Pt (IV) is incorporated into the structure of DDSs, which can then be reduced in the presence of GSH to Pt (II). This leads to the release of the platinum-based drugs and the disintegration of the DDS [30,87]. Platinum-based (Pt-based) complexes have the added advantage of exhibiting anticancer properties themselves; hence, they can be simultaneously used as prodrugs and as a redox-responsive unit in drug delivery systems [88]. An example of a Pt-based prodrug used in cancer treatments is Pt(IV) Cisplatin which, upon reduction to Pt(II) Cisplatin, releases free cisplatin to the tumor [30]. He et al. [30] developed a redox and pH dual sensitive micelle by reacting octahedral Pt(IV) cisplatin functionalized with two carboxylic acid groups [Pt(NH_3_)_2_Cl_2_(OOCCH_2_CH_2_CO_2_H)_2_] with an orthoester monomer [2,2′-((4,4′-(Oxybis(methylene)) bis(1,3-dioxolane-4,2-diyl)) bis(Oxy)] to form the polymeric micelle backbone. The micelle was then loaded with DOX, at a high capacity and efficiency, for enhanced anti-tumor efficacy via simultaneous delivery of Pt(II) cisplatin and DOX. In vitro studies used dithiothreitol (DTT) as a reducing agent and found that the reduction of Pt(IV) in the micelle led to over 80% release of both drugs within 9–12 h at a pH of 5.0, mimicking tumor microenvironments. The micelles also exhibited longer circulation times, allowing for higher accumulation at the tumor site via the enhanced permeation retention effect (EPR), were stable, and had minimal side effects on other organs [30]. Hence, this redox-responsive Pt-based micelle exhibited excellent properties as an effective and rapid drug delivery system for cancer treatment.

In addition to being incorporated into the backbone of micelles, platin conjugations have also been utilized to form redox-sensitive nanoparticles for drug delivery [89,90]. Ling et al. [89] utilized an octahedral Pt(IV) prodrug and coated it with lipid-PEG to form a biocompatible nanoparticle. Upon exposure to GSH, Pt(IV) is reduced to square planar Pt(II) and the nanoparticle disintegrates, releasing the Pt(II)-based cisplatin at the tumor site. The nanoparticle was found to have enhanced circulation time and better accumulation at the tumor site, allowing for successful drug delivery with fewer side effects [89]. Meanwhile, Wang et al. [90] integrated platin conjugation into nanoconjugates made up of a gamma-polyglutamic acid backbone and citric acid side chains, where octahedral succinic acid axially-functionalized Pt (IV) cisplatin was conjugated to the side chains. The nanoconjugate was dually responsive to pH and GSH, where the polymer underwent hydrolysis due to low pH, then the Pt (IV) group was reduced to Pt(II) cisplatin by GSH. In vitro and in vivo studies showed the nanoconjugate DDS to have excellent anti-tumor activity while also exhibiting significantly lower toxic side effects [90]. Thus, platin conjugation is another useful redox-responsive moiety that allows for the design of redox-responsive DDSs as well as Pt-based prodrugs. Table 1 below summarizes all the chemical moieties used for the design and synthesis of redox-responsive DDSs, along with their reduction reactions.

## 4. Redox Responsive DDSs with Disulfide Bonds

As mentioned earlier, disulfide bonds are among the most popular and widespread chemical moieties incorporated into redox-responsive DDSs. In fact, disulfide groups have been incorporated into different types of nanocarriers, such as polymeric micelles [38,39,44,59,60,62,63,65,66,67,68,69,110,111,112,113,114,115], liposomes [7,91,92,93,94,95,96,97,116], nanogels [64,98,99,100,101,102,103,104,105,106,107,108,109,117,118,119,120,121,122], hydrogels [123], mesoporous silica nanoparticles [61], metal-organic-frameworks [124], lipid nanoparticles [58], and hybrid DDSs combining two or more of these nanocarriers [32,125], among others. This section will herein review and focus on disulfide-containing polymeric micelles, liposomes, and nanogels due to their widespread nature and favorable properties.

### 4.1. Polymeric Micelles

Polymeric micelles often consist of copolymers with amphiphilic character (hydrophilic and hydrophobic character); these polymers self-assemble in solution to form a micelle, where the hydrophilic moieties form the outer shell and hydrophobic moieties form the inner core [87]. Micelles are highly advantageous DDSs as they can encapsulate hydrophobic drugs, that normally have low biocompatibility, to make them more biocompatible, hence allowing for efficient drug delivery with reduced side effects [126]. Disulfide linkers and crosslinkers have been extensively incorporated into polymeric micelles to make them redox-responsive through different compounds, such as cystamine [60,69], L-cysteine [113], cysteamine hydrochloride [114,115], lipoic acid [39,67], pyridylsulfide (PDS) [65], dithiobismaleimideoethane (DTME) [38], bis(ethylene acrylate) disulfide, also known as disulfide based diacrylate [59], 3,3′-dithiodipropionic acid (DPA) [63,68], 3,3′-dithiodipropionic anhydride (DPAH) [44], dithiodipropionic chloride [111], 2,2′-dithiodiethanol [110], 2-mercaptoethanol [112] and disulfide based dimethacrylates (DSDMA) [66], among many others.

Moreover, redox-responsive polymeric micelles have been generally synthesized with synthetic polymers. For example, Le et al. [38] utilized poly(ethylene oxide)-*block*-(furfuryl methylacrylate) (PEO-*b*-PFMA) block copolymers to prepare self-assembled micelles in the presence of disulfide-containing DTME core crosslinkers. The PEO-*b*-PFMA polymers were synthesized through a reversible addition-fragmentation chain transfer (RAFT) polymerization reaction, followed by micelle self-assembly, doxorubicin (DOX) loading, and disulfide core crosslinking through a click-Diels Alder reaction at 60 °C. In vitro studies in the presence of DTT as a reducing agent revealed a burst release of over 50% of the encapsulated DOX within 96 h, attributed to disulfide reduction and micelle disintegration. Meanwhile, minimal release was observed when DTT was absent, indicating successful targeted delivery and release only at the tumor site. Additionally, cytotoxicity studies using non-cancerous human embryonic kidney HEK 293 cells revealed excellent biocompatibility of the micelles and minimal cytotoxicity at concentrations as high as 500 µg mL^−1^. However, studies on human hepatocellular carcinoma HepG2 cell lines revealed the high anti-tumor activity of DOX-loaded PEO-*b*-PFMA-based micelles [38]. Zhang et al. similarly synthesized core-crosslinked polymeric micelles through RAFT polymerization followed by DTT treatment to induce disulfide crosslinking (using PDS) for curcumin delivery [65]. The copolymer was folic acid-coated PEG-PDS, and the loaded micelle exhibited high cytotoxicity towards a GSH-treated human Henrietta Lacks (HeLa) strain cancer cell line as well as stable, prolonged blood circulation and excellent tumor accumulation [65]. However, it is important to note that one common limitation of crosslinked micelles is that the enhanced stability induced by cross-linkage also leads to relatively slower DOX release [38]. Nonetheless, these redox-responsive micelles proved excellent in targeted drug release.

Duan et al. also developed a synthetic-based reduction-responsive micelle via a one-pot synthesis scheme to deliver DOX [59]. The amphiphilic polymer was synthesized by first linking DOX to 3,3′,4,4′-Diphenylsulfonetetracarboxylic Dianhydride (DSDA), containing the disulfide linker, via a Michael reaction. This was followed by the addition of amino-functionalized PEG to form the DOX-DSDA-PEG amphiphilic polymer, with PEG acting as the hydrophilic shell and DOX-DSDA as the hydrophobic core. The developed micelles were nontoxic, had a rapid accumulation and distribution at the tumor site, and had high DOX release rates at GSH concentrations of 1 mg mL^−1^, while also being highly toxic to the A549 human non-small cell lung cancer cell line [59]. Meanwhile, Wang et al. developed a triblock copolymer using PEG, poly(ε-caprolactone) (PCL), and poly(2,4-dinitrophenylthioethyl ethylene phosphate) (PPE, a polyphosphoester), where PPE is the middle segment modified with 2-mercaptoethanol to contain terminal thiol groups: PCL-*b*-PPE_SH_-*b*-PEG [112]. The terminal thiol groups, upon self-assembly into core-shell-corona micelles, react with each other to form disulfide cross-linkages in the shell. These micelles were effective in loading DOX and releasing it in GSH environments, in addition to being biocompatible and highly cytotoxic to A549 cells, proving to be a potential redox-responsive DDS for cancer applications [112].

Polymeric micelles with disulfide shell-cross-linkages, developed by Wang et al., have also been found to be effective in proliferating A549 cancer cells, as well as human breast cancer cell line MCF, via effective DOX delivery [69]. These micelles were based on *N*-acetyl glucosamine-poly(styrene-alt-maleic anhydride)_58_-*block*-polystyrene_130_ synthetic polymers and responded to dual pH and redox stimuli, with efficient intracellular drug delivery through energy-dependent and receptor-mediated endocytosis mechanisms [69]. Similarly, Luo et al. developed dual pH and redox responsive micelles for DOX delivery; however, this was done using varying mixtures of two synthetic, poly(*β*-amino esters) (PAE) based copolymers, one that was pH sensitive (mPEG-*b*-PAE) and one that was redox-sensitive with disulfide cystamine sidechain linkers (PAE-SS-mPEG) [60]. While all micelle compositions had cell viabilities exceeding 95% when unloaded, the polymeric micelles with double the concentration of PAE-SS-mPEG copolymers exhibited the greatest morphological change upon reduction with DTT, fastest DOX release rates, and highest cumulative DOX release in in vitro studies using HepG2 cell line [60]. Zhuang et al. also developed dual pH- and redox-responsive micelles using the synthetic polymers mPEG and polymeric tetraphenylethylene-*co*-2-azepane ethyl methacrylate [mPEG-P(TPE-*co*-AEMA) [110]. 2,2-dithioldiethanol was used to introduce disulfide bonds as linkers between TPE and AEMA in the backbone. These micelles had a high DOX loading efficiency, prolonged circulation, and excellent anti-tumor efficacy in vitro and in vivo, with no significant adverse effects [110].

While polymeric micelles based on synthetic polymers are common, recent studies have shifted into using biopolymers for micelle-based DDSs due to their better biocompatibility, organized structures, higher drug loading capacity, and nontoxicity [87,127]. An example of an efficient, redox-responsive biopolymer-based micelle recently developed is hydroxyethyl chitosan (HECS) linked with n-octylamine (OA) through a disulfide bond (derived from DPAH) (HECS-ss-OA) micelle coated with hyaluronic acid for specific tumor targeting [44]. The HECS-ss-OA micelle was synthesized through a complex multistep synthetic scheme and loaded with gambogic acid, a naturally derived chemical agent that can induce apoptosis of cancer tumors, among other anticancer activities [44,128]. The hyaluronic acid coating was found to increase the in vitro and in vivo biocompatibility of the micelles, as confirmed by the rapid gambogic acid release in the presence of reducing agents, as well as the increased targeted anti-tumor efficacy, compared to the control that lacked disulfide bonds [44]. Anh et al. also developed chitosan-based redox-responsive micelles, where disulfide linkages were incorporated in the polymeric backbone via lipoic acid conjugation with low molecular weight water-soluble chitosan (LMWSC) [39]. These micelles had over 80% cell viability in HeLa cultures, indicating biocompatibility and non-toxicity. Furthermore, these micelles had high DOX loading capacities with high release rates in GSH and rapid biodistribution, where the DOX-loaded micelles took only 4 h to enter the nucleus [39]. Lee et al. similarly designed chitosan-based micelles; however, the micelles were developed using chitosan modified with lactose (Chitlac), which was conjugated with disulfide containing Pluronic among other groups to induce pH and redox sensitivity. These micelles successfully entrapped and delivered Taxol, with Taxol-loaded micelles exhibiting high cytotoxicity and anti-tumor efficacy [62]. Therefore, chitosan biopolymers and their derivatives serve as good, biocompatible polymers for the synthesis of redox-responsive polymeric micelles for drug delivery.

Polynucleotides are another class of biopolymers that have been integrated into the design of redox-responsive micelles for the delivery of anti-tumor agents. Zhang et al. used a series of RAFT polymerization reactions to develop a methacryloyluridine-based micelle. In their work [66], poly(PEG methyl ether methacrylate) [P(PEGMEMA)] was first synthesized via a RAFT polymerization, then chain extension with 5′-O-methacrloyluridine (MAU) was executed via another RAFT polymerization, and, after micelle self-assembly, a third RAFT polymerization reaction was utilized to induce core-disulfide crosslinks using bis(2-methacryloxyethyl) disulfide (DSDMA). The resulting micelle was unique in its ability to load hydrophilic drugs, such as the model Vitamin B2 drug, due to its hydrophilic core, which is atypical as micelle cores are generally hydrophobic. These micelles were also found to be nontoxic to COS-1 cells and underwent rapid degradation in approximately 40 min when exposed to 0.65 M DTT; however, further studies are important to determine their sensitivity to GSH and anti-tumor efficacy in vivo [66]. Phosphorylcholine (PC), a small amphiphilic biomolecule derived from phospholipids, has also been used as a biocompatible building block for micelles, although commonly used for liposome development. Li et al. functionalized PC with lipoic acid, containing disulfide linkers, to form di-lipoyl-glycerophosphorylcholine (di-LA-PC) amphiphilic molecules that can self-assemble into micelles and encapsulate paclitaxel (PTX) [67]. The di-LA-PC-based micelles were highly biocompatible in MCF-7, A549, and HrGp-2 cancer cell lines, while the PTX-loaded micelles exhibited high efficiency in inhibiting tumor growth in vivo for mice injected with 4T1 mammary carcinoma cells [67]. Therefore, polynucleotides are also useful in the synthesis of redox-responsive DDSs.

Moreover, polypeptides have also been utilized in the development of disulfide-containing redox-responsive DDSs. Ding et al. [115] functionalized methoxyl PEG (mPEG) with a disulfide bond (using cysteamine hydrochloride) and terminal amino group to form mPEG-SS-NH_2_, followed by a ring-opening polymerization reaction with poly(ε-benzyloxycarbonyl-L-lysine) (PZLL), the polypeptide, to form the block copolymer that later self-assembles into micelles. These mPEG-SS-PZLL micelles successfully loaded DOX with a 30 wt% efficiency and exhibited very good hemocompatibility and biocompatibility. Furthermore, these micelles rapidly degraded in GSH and showed excellent cytotoxicity to HeLa and HepG2 cells [115]. Similarly, Wen et al. also developed mPEG-SS-PZLL nanomicelles for DOX delivery, with a lower loading efficiency of 16.7 wt%, good sensitivity to GSH, and high cytotoxicity towards MCF-7 cancer cells [111]. Qu et al. [114] also developed a polypetptide, S–S containing micelle using cysteamine hydrochloride to form the disulfide-containing N,N′-methylene-bis-acylamide (BACy) crosslinking agent, poly (γ-benzyl-L-glutamate) (PBLG) as the polypeptide monomer, and *N*-isopropylacrylamide (NIPPAM) as the comonomer. Radical copolymerization, with the initiator 2,2-azobisisobutyronitrile (AIBN), was used to form the polymeric backbone, which later self-assembled into micelles capable of encapsulating DOX with a 56% efficiency. The DOX-loaded micelle exhibited rapid disintegration and DOX release in GSH environments, as well as being sensitive to multiple stimuli, including GSH, pH, and temperature [114]. Moreover, low molecular weight protamine (LMWP), a polypeptide, was also used in the development of redox-responsive micelle [113]. In their study, LMWP-Vitamin E succinate disulfide crosslinked micelles were developed for the codelivery of DTX and microRNA-4638-5p. These micelles showed synergistic effects and excellent anti-tumor efficacy in vitro and in vivo, proving a potential DDS for clinical applications [113]. In addition to the studies mentioned above, other biopolymers utilized for the synthesis of redox-responsive micelles include starch [129], dextran [68], hyaluronic acid [130], xylan [63], and others. Hence, disulfide bonds can be successfully integrated into micelles as linkers and crosslinkers using synthetic polymers and biopolymers to allow for efficient, targeted delivery of anti-tumor agents. Table 2 below summarizes disulfide-containing redox-responsive polymeric micelles reported in the literature.

### 4.2. Liposomes

Liposomes are spherical nanocarriers composed of one or more phospholipid bilayers, consisting of hydrophilic heads and hydrophobic tails [87,131]. The presence of both hydrophilic and hydrophobic regions in liposomes renders them more valuable because it allows liposomes to carry and deliver lipophilic, hydrophilic, and amphiphilic drugs [131,132]. Despite liposomes being one of the first treatments to reach clinical applications, redox-responsive liposomal drug delivery for cancer treatment is less explored in the literature [131]. Disulfide bonds have been incorporated into various liposomes to facilitate redox-responsive anti-tumor drug delivery in numerous studies, where they are either a part of the phospholipid layer or used to conjugate molecules to the liposomal surface [7,91,92,93,94,95,96,97,116,133]. He et al. [93] designed a redox-responsive liposome drug delivery system for clinical use and application by conjugating camptothecin (CPT) (an anticancer agent targeting breast cancers) with glyceralphosphorylcholine (GPC) through disulfide linkages to create CPT-ss-3-GPC and CPT-ss-11-GPC, which were assembled into liposomes via reverse evaporation. The disulfide linkages were incorporated using a disulfide-thiol exchange reaction between 3-(tritylthio) propionic acid with either 3- or 11- carbon chain lengths and 2-(pyridyl-disulfanyl)ethanol. Exposure to GSH at different concentrations and durations revealed that the drugs release speed and quantity increased with increasing GSH concentration, as ~90% of CPT was released within 20 min at 20 mM GSH. Moreover, both liposomes were hemocompatible and biocompatible, but CPT-ss-11-GPC has the best cytotoxicity to MCF-7 cells and anti-tumor efficacy in vivo as tumor inhibition was approximately 64%. Hence, the liposome responded well to tumor-reducing environments with high sensitivity and has the potential to be used in clinical trials in addition to being used for co-delivery of multiple drugs, not just CPT [93].

Moreover, Du et al. developed a redox-responsive alternative to PC for liposome synthesis, PC hydrophobic chains were integrated with disulfide bonds through a multi-step synthetic scheme [116]. First, dithiohydroxypropyl PC is synthesized by thiolating the GPC end using 3-(tritylthio)propionic acid. Then, the hydrophobic phospholipid chains, 2-(dodecyldisulfanyl) pyridine are synthesized to contain disulfide bonds by reacting 1-dodecanethiol with 1,2-di(pyridine-2-yl)disulfane. Finally, the thiol-ended dithiohydroxypropyl PC is reacted with the disulfide containing 2-(dodecyldisulfanyl) pyridine through a disulfide-thiol exchange reaction to form the final, S–S containing redox responsive phosphatidylcholine (SS-PC) [91,116]. In a follow up study by Du et al., these SS-PCs were combined with cholesterol and 1,2-diastearoyl-sn-glycero-3-phosphoethanolamine-PEG_2000_ to assemble liposomes using the reverse evaporation method that can efficiently load and deliver PTX [91]. The SS-PC-based liposomes exhibited longer circulation duration due to the stealth PEG-coating that allowed these liposomes to dodge the reticuloendothelial system. Furthermore, the PTX-loaded liposomes showed rapid drug release in reducing environments and improved anti-tumor activity in vitro and in vivo. Wang et al. used the same SS-PC developed by Du et al. for the development of liposomes for irinotecan delivery, where a solvent (ethanol) injection method was used to assemble liposomes from SS-PC chains, 1, 2-Distearoyl-sn-glycero-3-phosphoethanolamine-Poly(ethylene glycol) (DSPE-PEG_2000_), cholesterol, and 1,2-distearoyl-sn-glycero-3-phosphocholine (DSPC) [92]. These liposomes were biocompatible, with significantly high drug loading (~32%) and encapsulation efficiencies (~98%) and had increased rates of internalization as well as excellent drug release upon 1 mM GSH exposure (~60% release in 24 h) [92].

Other reduction-sensitive liposomes assembled via the solvent injection method include those synthesized by Yin et al. [95] and Chi et al. [96] for DOX delivery and osteosarcoma cancer therapy. Yin et al. prepared disulfide-containing chitooligosaccharide (COS) chains and mixed them with DSPE-PEG_2000_-estrogen chains to assemble estrogen functionalized liposomes with COS attached to the liposomal surface through the disulfide linker, DPA. When introduced to a reducing condition, COS was released and the liposome degraded to release DOX rapidly. Furthermore, the liposomes exhibited enhanced osteosarcoma targeting through estrogen functionalization, as demonstrated by the higher toxicity to MG63 osteosarcoma cells compared to the normal liver L02 cells [95]. Meanwhile, Chi et al. [96] functionalized their Cholestrol-SS-PEG_2000_-based liposomes with hyaluronic acid, a ligand to the CD44 significant osteosarcoma target molecule. Similar to Yin et al. [95], the disulfide linkers were incorporated using DPA and the DOX-loaded liposomes exhibited rapid burst DOX release in reducing environments (10 mM GSH). The DOX-loaded liposomes were also highly cytotoxic to osteosarcoma MG62 cells and had high tumor efficacies in MG63-inoculated BALB/c nude mice [96]. Hence, these redox-responsive liposomes serve as potential clinical agents for osteosarcoma cancer therapies.

Moreover, film methods, more conventional methods for liposomal assembly, have also been proved to be useful for the development of redox-responsive liposomal DDSs. Wang et al. synthesized PTX-disulfide-linked lysophosphatidylcholine through a 5-step conjugation synthetic scheme, where disulfide bonds were incorporated using 2-hydroxyethyl disulfide [94]. The PTX-SS-lysophosphatidylcholine was mixed with cholesterol, an EPC ligand, and mPEG_2000_-DSPE to form liposomes using the dry film method. The resultant liposomes exhibited high dissociation rates and PTX release upon reduction in addition to high cytotoxicity towards MCF-7 and A549 cell lines [94]. On the other hand, Peng et al. assembled redox-responsive liposomes using thin film hydration for co-delivery of DOX and lonidamine (LND) [7]. The liposome was mainly composed of PEGylated cholesterol molecules with disulfide linkages (originating from DPA) and modified with glucose and triphenylphosphonium (TPP) for enhanced tumor targeting, larger interference towards mitochondrial functions, and improved delivery across the brain–blood barrier to reach glioma tumors. The co-loaded liposomes exhibited high anti-tumor efficacies in C6-bearing Kunming mice and superior pharmacokinetic properties, with synergistic effects of DOX and LND delivery [7]. Thus, film methods are also useful for redox-responsive liposomal assembly.

All previously discussed liposomes required extra steps for their assembly, which is generally the case for liposomes as they do not tend to self-assemble like micelles. However, Liu et al. were able to develop unique redox-responsive phospholipids that could self-assemble into liposomes [97]. To synthesize the lipid, 3-[(2-Aminoethyl) dithio] propionic acid (MPA-SS-NH2) was conjugated with Pyropheophorbide-a (PPa) to form MPA-SS-PPa, which was subsequently mixed with 1-palmitoyl-2-hydroxy-sn-glycero-3-phosphocholine (Lyso-PC), 4-(dimethylamino)pyridine (DMAP), and EDC and stirred for 12 h, with other chemical reagents, to form the redox response-lipids. These lipids self-assembled into liposomes that efficiently encapsulated 1-methyl tryptophan (NLG-8189), an indoleamine-2,3-dioxygenase inhibitor (IND), which was released rapidly upon 10 mM GSH exposure due to disulfide bond cleavage and liposome disintegration. These loaded-liposomes were cytotoxic to 4T1 cells and were dually activated by light, allowing for photoimmunotherapy, exhibiting excellent characteristics for potentially treating metastatic tumors [97]. Thus, different types of redox-responsive liposomes containing disulfide bonds have been synthesized and have exhibited excellent properties for cancer treatment. Table 3 below summarizes disulfide-containing redox-responsive liposomes reported in the literature.

### 4.3. Nanogels

Nanogels are becoming increasingly popular frameworks for redox-responsive DDSs and are composed of polymers crosslinked together to form a solid-like gel material with hollow spaces that can encapsulate therapeutic agents. Disulfide linkers can be used to directly link therapeutic agents to the nanogel framework [109] or to crosslink the polymeric backbones, which is more common [64,98,99,100,101,102,103,104,105,106,107,108]. When exposed to GSH-reducing environments of tumors, these disulfide bonds disintegrate, chemically breaking up the nanogel and releasing the drugs. Numerous synthetic methods have been utilized to incorporate disulfide crosslinking in nanogel preparation. Five main synthetic pathways used to prepare redox-responsive nanogels include radical polymerization [98,99,100,101], ring-opening polymerization [103], Michael addition polymerization [104], branched arm crosslinking [105], and self-crosslinking [64,102,106,108], which are reviewed thoroughly in [134].

Radical polymerization generally consists of electrophilic or nucleophilic organic free radicals (consisting of an unpaired electron) that react together through four phases: initiation, propagation, transfer, and termination [135]. Radical polymerization reactions are of different types. Elkassih et al. utilized an oxidative radical polymerization reaction to synthesize redox-sensitive nanogels from (ethylenedioxy)diethanethiol (EDDET) monomers and disulfide-containing pentaerythritol tetramercaptoacetate (PETMA), in the presence of triethylamine [98]. The nanogels were further modified with Pluronic-127 for added stability. The resultant nanogels were biocompatible, exhibited rapid dissociation in reducing environments, and were effective in encapsulating the model dye Rhodamine B [98]. Conversely, Tian et al. mixed oligo(ethylene glycol) methacrylate, 2-(2-methoxyethoxy) ethyl methacrylate, and N,N′-bis(acryloyl)cystamine (BAC) (the S–S containing crosslinker) to form nanogels via an in situ free radical copolymerization reaction, which were later loaded with DOX [99]. The DOX-loaded nanogels exhibited rapid DOX release in 10 mM GSH (92.2% release in 48 h) and greatly inhibited HeLa cell growth [99]. Chen et al. and Li et al. also utilized radical polymerization reactions to form nanogels, where BAC was the disulfide-containing crosslinker [100,101]. In addition to being redox-sensitive, Chen et al. designed their nanogels to be photo- and pH-responsive [100], while Li et al. designed their nanogels to be temperature-sensitive [101]. Similarly, Li et al. [122] also designed dual temperature- and redox-responsive nanogels using radical polymerization mediated with cobalt, where DPA was used as the S–S containing crosslinking agent. Interestingly, the nanogel was formed by first synthesizing the poly(vinyl alcohol)-b-poly(*N*-vinylcaprolactam) (PVOH-*b*-PNVCL) copolymer, followed by micelle formation. The resultant micelles were converted into nanogels by lowering the temperature to below the volume phase transition temperature of the copolymer. These nanogels had good biocompatibility and high cytotoxicity toward MEL-5 cancer cells and mouse fibroblast-like L929 cells [122]. Moreover, methacrylated hyaluronic acid and cystamine bisacrylamide (CBA) have been reacted together through a radical polymerization to form S-S crosslinked nanogels with enhanced tumor targeting, rapid degradation in GSH, and high cytotoxic and apoptotic activity against H22 and LNCaP cell lines in vitro and in vivo [120]. Therefore, radical polymerization reactions are versatile synthetic methods suitable for designing and preparing redox-responsive nanogels.

Ring-opening polymerization (ROP) is another useful synthetic pathway that has been used for the integration of disulfide crosslinkers in nanogel assembly. As its name suggests, ROP reactions involve the opening of cyclic monomeric units to react with terminal ends of other monomers to form polymeric chains and are of many types [136]. ROP is especially useful in the synthesis of polypeptide-based redox-responsive nanogels. For example, Xing et al. developed redox-sensitive nanogels using L-cystine as a disulfide source through a simple, one-step ROP reaction between L-cystine *N*-carboxyanhydride (LC-NCA) and benzyl L-glutamate *N*-carboxyanhydride (BLG-NCA) using NH_2_-terminated PEG methyl ether (mPEG_1900_-NH_2_) as an initiator [103]. The biocompatible nanogels were loaded with indomethacin, and upon 10 mM GSH exposure, exhibited a burst release (~7 h.) followed by a sustained release where ~100% release was achieved after 200 h [103]. Similarly, He et al. [119] and Guo et al. [118] developed L-cystine crosslinked nanogels by a ROP reaction between LC-NCA and L-phenylalanine NCA (LP-NCA). However, He et al. used mPEG-NH_2_ as the initiator to form mPEG-P(LP-*co*-LC) nanogels for DOX delivery and prostate cancer treatment [119]. Meanwhile, Guo et al. used allyloxyl PEG-NH_2_ (aPEG-NH_2_) as the initiator to form aPEG-P(LP-*co*-LC) then further modified the nanogel to add nine arginine residues (R_9_) and form R_9_-PEG-P(LP-*co*-LC) for delivery of 10-hydroxycamptothecin (HCPT) and treatment of bladder cancer [118]. Both nanogels showed excellent redox-responsiveness and high tumor growth suppression in vitro and in vivo [118,119]. On the other hand, Jing et al. used the ROP synthetic pathway to form 2,3-dimethylmaleic anhydride poly(Z-L-lysine-*co*-L-cystine) (DMA-PLL-LC) nanogels of a core–shell structure, where the shell is pHe- (tumor microenvironment pH) sensitive and the core is GSH-sensitive thus leading to a stepwise disintegration process [117]. These nanogels had a high DOX loading capacity, excellent biocompatibility, and high cytotoxicity to HepG2 cells, thus making them promising candidates for clinical chemotherapy [117]. Hence, ROP is a useful synthetic pathway to induce disulfide crosslinking in nanogel-based DDSs.

Furthermore, Michael-addition polymerization is another useful reaction for nanogel synthesis, involving the reaction of amines with vinyl groups to form polymeric chains. Cheng et al. incorporated disulfide crosslinkers into nanogels through a Michael addition polymerization reaction between 4-(aminomethyl) piperidine (AMPD) and BAC (1:2 ratio), followed by excessive AMPD treatment to convert vinyl groups to amines, PEG conjugation, and thiol-disulfide exchange reactions to induce crosslinking of polymers and nanogel formation [104]. Meanwhile, branched-arm crosslinking is another synthetic route involving crosslinking branched, multi-armed PEG derivatives or other multi-armed polymers with disulfide bonds to form redox-responsive nanogels [134]. An example of nanogels developed through branched-arm crosslinking are those developed by Mandal et al., where a polyacrylic-based, 4-armed block copolymer was synthesized and crosslinked with 2-hydroexyethyl disulfide [105]. More specifically, the branched polymer used was pentaerythritol-poly(ε-caprolactone)-*block*-polyacrylic (Pe-PCL-*b*-PAA), and the disulfide crosslinker was incorporated via an esterification reaction between the carboxylic acid groups of the branched copolymer and the hydroxy groups of the crosslinker. The resulting nanogels effectively encapsulated and delivered DOX in GSH-reducing environments [105].

**Figure 5:**
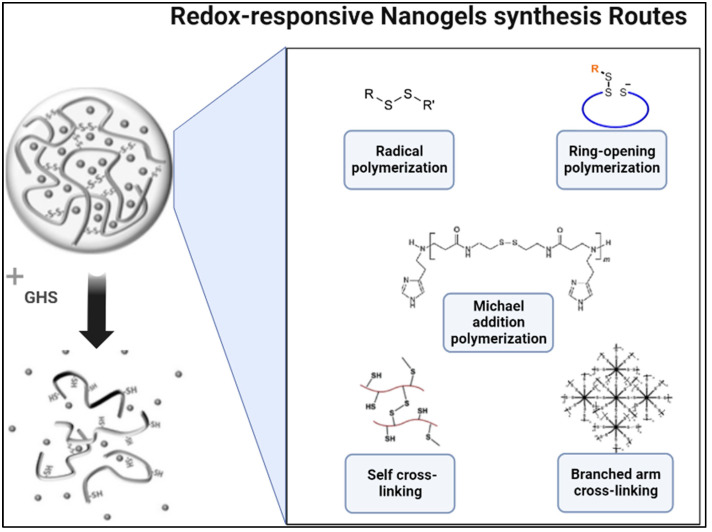
Synthetic routes for S–S crosslinking in redox-responsive nanogels.

Self-crosslinking is the most common synthetic pathway for nanogel formation. Through this pathway, disulfide crosslinking can be achieved through the conjugation of disulfide crosslinking agents onto the polymeric chains or through disulfide bond formation between thiol groups on the polymeric chains. For example, Cao et al. synthesized furfuryl amine and hydrazine-dual functionalized poly(styrene-alt-maleic anhydride) (PSM) polymer and then attached dithio-bis-maleimdoethane (DTME) (the disulfide crosslinker) to furfuryl amine on the polymers through a Diels–Alder reaction [106]. This Diels–Alder reaction led to cross-linkage of the nanogel through the DTME crosslinker, where each end of DTME was bonded to a different polymeric chain. These nanogels were further conjugated with DOX, and in vitro studies have shown excellent inhibition rates towards HepG2 cells as well as good GSH sensitivity. A similar method was used by Peng et al.; however, the disulfide crosslinker was attached to the polymeric chains through a Michael addition reaction [64]. The disulfide crosslinker used was synthesized from the esterification of dithiodiglycolic acid with DMAP and was attached to a hydrolyzed, NH_2_-containing poly(*N*-vinylpyrrolidone-co-*N*-vinylformamide) chain. These nanogels had good biocompatibility, rapid degradation in GSH, high DOX loading capacity, and DOX-release rates that increased with decreasing crosslinking densities [64]. Other disulfide crosslinker integrated via self-crosslinking include cystamine [108,121], cystamine tetra-hydrazine [102] and 3,3′-dithiodipropionic acid bis(*N*-hydroxysuccinimide ester (DTSP) [107], all of which led to the formation of redox-responsive nanogels for anti-tumor drug delivery. Figure 5 summarizes the synthetic routes for incorporation of disulfide crosslinking in nanogels.

All the synthetic pathways discussed above utilize disulfide bonds as crosslinkers in nanogels. However, disulfide bonds have also been used as linkers in the polymeric backbone to synthesize redox-responsive nanogels. For example, Qu et al. synthesized CPT monomers containing disulfide bonds by reacting CPT with 2-((2-hydroxyethyl) disulfanyl) ethyl methacrylate (HSEMA) [109]. This CPT monomer containing a disulfide bond is then mixed with methacrylic acid (MAA) and N,N′-methylenebisacrylamide (Bis) (as a crosslinker) and reacted via a distillation–precipitation polymerization reaction to form the redox-sensitive nanogel prodrug. The nanogel was found to have good tumor inhibiting behavior in HepG2 (in vitro) cells and HepG2-tumor-bearing BALB/c mice (in vivo), with negligible side effects and excellent release in 10 mM GSH reducing environments [109]. Hence, disulfide bonds are useful as both linkers and crosslinkers in the development of redox-responsive nanogels for drug delivery. Table 4 summarizes disulfide-containing redox-responsive nanogels reported in the literature.

## 5. Conclusions and Recommendations

In conclusion, redox-responsive drug delivery systems are an emerging field of nanomedicine that have great potential to be utilized in clinical applications for cancer treatment. Elevated GSH levels in tumor microenvironments create a reducing environment that allows for site-specific targeting, preventing the death of surrounding non-cancerous cells and reducing side effects on major organs, unlike standard chemotherapy procedures [3,17,33]. Several chemical groups have been utilized in drug delivery systems to introduce redox sensitivity, such as disulfide bonds, diselenide bonds, succinimide-thioether linkages, tetrasulfide bonds, and platin conjugation [17,33,43,54]. These have been incorporated into different nanocarriers, with liposomes, polymeric micelles, and nanogels being the most common carriers [87].

Despite the recent major advancements in this field, further research is needed for these drug delivery systems to proceed through clinical trials and enter the market. For instance, studies on redox-responsive drug delivery systems using diselenide bonds, succinimide-thioether linkages, and platin conjugation are mostly limited to micelles and nanogels. Further research on their integration into liposomes, which have demonstrated excellent therapeutic effects in clinical trials, could introduce more efficient, relevant, and applicable SDDSs. Furthermore, the integration of two or more of these chemical entities together in one system, such as a combination of disulfide and diselenide bonds as linkers, could also be explored as a potentially more successful system with rapid, targeted drug delivery to tumors. With continuous research into these redox-sensitive materials, simple and effective cancer treatment approaches can eventually be developed to improve the lives of millions of cancer patients and reduce the global impact of cancer.

## Figures and Tables

**Table 1:** Summary of chemical entities used for the synthesis of redox-responsive DDSs.

Reduction Sensitive Moiety	Chemical Structure	Structure after GSH Reduction	Studies Utilizing These Moieties
Disulfide	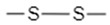	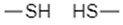	[7,32,38,39,44,59,60,61,62,63,64,65,66,67,68,69,91,92,93,94,95,96,97,98,99,100,101,102,103,104,105,106,107,108,109]
Diselenide	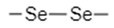	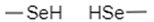	[29,46,72,73,74,75,76,77,79,80,81,82,83,84]
Succinimide-thioether	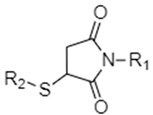	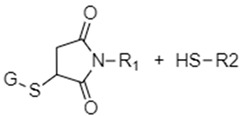	[8,28,47,48,49,50,51,52]
Tetrasulfide	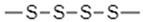	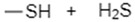	[53,54,55,56]
Platin conjugation	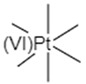 octahedral	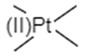 square planar	[30,89,90]

**Table 2:** Summary of disulfide-based redox-responsive polymeric micelles.

Micelle Components	S-S Source	S-S Position	Payload	Biological Evaluation	Ref.
LMWSC, Lipoic acid	Lipoic acid	Linker (backbone)	DOX	HEK293, HeLa, AGS (in vitro)	[39]
Chitlac, Pluronic-SS-NH_2_, spiropyran/boronic acid conjugated poly(dimethylamino ethyl methacrylate-co-methacrylic acid)	Pluronic-SS-NH_2_	Linker (2 moieties)	Taxol	MDCK, KB (in vitro)	[62]
di-LA-PC,	Lipoic acid	Crosslinker (core)	PTX	MCF-7, A549, HrGp-2 (in vitro), BALB/c mice with 4T1 mammary carcinoma cells (in vivo)	[67]
P(PEGMEMA), MAU, DSDMA	DSDMA	Crosslinker (core)	Vitamin B2	COS-1 (in vitro)	[66]
Chitosan, hyaluronic acid,	DPAH	Linker (2 moieties)	Gambogic acid	A549 (in vitro), BALB/c mice with A549 tumors (in vivo)	[44]
Xylan, curcumin	DPA	Linker (2-moieties)	5-fluorouracil-stearic acid, curcumin	HT-29, HCT-15 (in vitro)	[63]
poly(gamma-benzoyl-L-glutamate)-*block*-dextran	DPA	Crosslinker (shell)	DOX	HeLa, HepG2 (in vitro)	[68]
mPEG, PZLL	Cysteamine hydrochloride	Linker (2 moieties)	DOX	HeLa, HepG2 (in vitro), BALB/c nude mice (in vivo)	[115]
mPEG, PZLL	dithiodipropionic chloride	Linker (2 moieties)	DOX	MCF-7 (in vitro)	[111]
BACy, AIBN, PBLG, NIPPAM	Cysteamine hydrochloride	Crosslinker (core)	DOX	HeLa, HUVEC (in vitro)	[114]
LMWP, Vitamin E succinate	L-cysteine	Crosslinker (shell)	DTX, microRNA-4638-5p	PC3, Du145 (in vitro), BALB/c mice bearing PC3 tumor (in vivo)	[113]
PEG, PCL, PPE	2-mercaptoethanol	Crosslinker (shell)	DOX	A549 (in vitro)	[112]
DOX, DSDA, and mPEG-NH_2_	DSDA	Linker (backbone)	DOX	A549 (in vitro)	[59]
PEO-*b*-PFMA	DTME	Crosslinker (core)	DOX	HEK293, HepG2 (in vitro)	[38]
mPEG-*b*-PAE and PAE-SS-mPEG	Cystamine	Linker (side chain)	DOX	HepG2 (in vitro)	[60]
folic acid-PEG-PDS	PDS	Crosslinker (core)	Curcumin	HeLa (in vitro), BALB/c with xenograft model of cervical cancer (in vivo)	[65]
*N*-acetyl glucosamine-poly(styrene-alt-maleic anhydride)58-*b*-polystyrene130	cystamine	Crosslinker (shell)	DOX	A549, MCF-7 (in vitro)	[69]
mPEG, P(TPE-*co*-AEMA)	2,2′-dithiodiethanol	Linker (backbone)	DOX	4T1 (in vitro), BALB/c mice bearing 4T1 tumors (in vivo)	[110]

**Table 3:** Summary of disulfide-containing redox-responsive liposomes.

Liposome Components	Disulfide Source	Payload	Biological Evaluation	Ref.
SS-PC, cholesterol, DSPE-PEG_2000_	3-(tritylthio)propionic acid in SS-PC	PTX	MCF-7, A549 (in vitro), 4T1 tumor-bearing BALB/c mice (in vivo)	[91]
SS-PC, DSPC, DSPE-PEG_2000_, Cholesterol	3-(tritylthio)propionic acid in SS-PC	Irinotecan	MCF-7, A549 (in vitro) 4T1 tumor-bearing BALB/c mice and SD rats (in vivo)	[92]
CPT-SS-GPC	3-(tritylthio)propionic acid of different chain lengths (3 and 11) & 2-(pyridyl-disulfanyl)ethanol	CPT	MCF-7, HepG2, A549 (in vitro) MCF-7 inoculated BALB/c mice (in vivo)	[93]
Chol-SPG, Glucose, Chol-TPP	DPA	DOX & LND	C6 and bEnd.3 (in vitro) C6-bearing Kunming mice (in vivo)	[7]
PTX-SS-lysophosphatidylcholine, cholesterol, EPC, mPEG_2000_-DSPE	2-hydroxyethyl disulfide	PTX	MCF-7, A549 (in vitro)	[94]
DSPE-PEG2000-Estrogen, Chol-SS-COOH, COS	DPA	DOX	MG63, L02 (in vitro) MG63 osteosarcoma inoculated BALB/c mice (in vivo)	[95]
Chol-SS-mPEG, Hyaluronic acid	DPA	DOX	L02, MG63 (in vitro) SD rats and MG63 inoculated BALB/c mice (in vivo)	[96]
MPA-SS-NH2, PPa, Lyso-PC, EDC, DMAP	AEDP	NLG8189	4T1 (in vitro) 4T1 breast cancer inoculated BALB/c mice	[97]

**Table 4:** Summary of disulfide-containing redox-responsive nanogels.

Nanogel Components	S-S Source and Position	S-S Cross-Linking Synthetic Route	Payload	Biological Evaluation	Ref.
EDDET, PETMA, TEA, Pluronic-127	EDDET and PETMA (crosslinker)	Radical polymerization	Rhodamine B dye	HeLa (in vitro)	[98]
2-(2-methoxyethoxy) ethyl methacrylate, oligo(ethylene glycol) methacrylate, BAC	BAC (crosslinker)	Radical polymerization	DOX	HEK-293T, HeLa (in vitro)	[99]
1′-(2-methacryloxyethyl)-3′,3′-dimethyl-6-nitro-spiro(2H-1-benzo-pyran-2,2′-indoline), acrylic acid, BAC	BAC (crosslinker)	Radical polymerization	DOX	MCF-7 (in vitro)	[100]
*N*-Isopropylacrylamide (NIPA), *N*-hydroxyethylacrylamide (HEAA), BAC	BAC (crosslinker)	Radical polymerization	Rhodamine 6G Dye, propranolol	L929 (in vitro)	[101]
PVOH-*b*-PNVCL	DPA (crosslinker)	Radical polymerization	Nile red	MEL-5, L929 (in vitro)	[122]
MAHA, CBA	CBA (crosslinker)	Radical polymerization	DOX	H22, LNCaP (in vitro), BALB/c mice injected with LNCaP, ICR mice injected with H22 (in vivo)	[120]
BLG-NCA, LC-NCA, mPEG_1900_-NH_2_	L-cystine (crosslinker)	ROP	indomethacin	HeLa (in vitro)	[103]
mPEG-P(LP-*co*-LC)	L-cystine (crosslinker)	ROP	DOX	RM-1 (in vitro), C57BL/6 mice injected with RM-1 cells (in vivo)	[119]
R_9_-PEG-P(LP-*co*-LC)	L-cystine (crosslinker)	ROP	HCPT	BC 5637 (in vitro), SD rats with orthotopic BC, C57BI/6 mice with orthotopic BC (in vivo)	[118]
DMA-PLL-LC	L-cystine (crosslinker)	ROP	DOX	HepG2 (in vitro)	[117]
AMPD, BAC, PEG	BAC (crosslinker)	Michael addition polymerization	FITC-dextran	-	[104]
Pe-PCL-*b*-PAA	2-hydroxyethyl disulfide (crosslinker)	Branched arm crosslinking	DOX	C6 glioma cells, fibroblast cells, HaCat cells (in vitro)	[105]
poly(*N*-vinylpyrrolidone-co-*N*-vinylformamide), DMAP, dithioglycolic acid	DMAP + dithiodiglycolic acid (crosslinker)	Self-crosslinking	DOX	HeLa (in vitro)	[64]
PSM, furfuryl amine, hydrazine, DTME	DTME (crosslinker)	Self-crosslinking	DOX	HepG2, HEK293 (in vitro)	[106]
2,6-diamino pyridine, uracil-functionalized poly(*p*-phenylenevinylene)	DTSP (crosslinker)	Self-crosslinking	DOX	HeLa (in vitro)	[107]
Hyaluronic acid, cystamine, gold nanorods (AuNRs)	Cystamine (crosslinker)	Self-crosslinking	DOX	MCF-7, drug-resistant MCF-7 ADR (in vitro)	[108]
Alginate, cystamine	Cystamine (crosslinker)	Self-crosslinking	DOX	CAL-72 (in vitro)	[121]
Xanthan, cystamine tetra-hydrazine,	Cystamine tetra-hydrazine (crosslinker)	Self-crosslinking	DOX	HeLa, 3T3 cells (in vitro)	[102]
MAA, CPT, Bis, HSEMA	HSEMA (linker)	-	CPT	HepG2 (in vitro) HepG2 tumor-bearing BALB/c mice (in vivo)	[109]

## Data Availability

Not applicable.
